# Does It Really Pay-Off? Comparison of Lymphadenectomy versus Observational Approach in Skin Melanoma with Positive Sentinel Node Biopsy: Systematic Review and Meta-Analysis

**DOI:** 10.3390/jcm11133880

**Published:** 2022-07-04

**Authors:** Karolina Richter, Tomasz Stefura, Krzysztof Macheta, Jonasz Tempski, Jakub Kazoń, Magdalena Szeremeta, Paweł Klimont, Marta Kołodziej-Rzepa, Tomasz Wojewoda, Wojciech M. Wysocki

**Affiliations:** 1 Faculty of Medicine and Health Sciences, Andrzej Frycz Modrzewski Krakow University, Gustawa Herlinga-Grudzińskiego 1, 30-705 Krakow, Poland; karolinaa.richter@gmail.com (K.R.); kmacheta9@gmail.com (K.M.); jonasz.tempski@op.pl (J.T.); kuba0185@wp.pl (J.K.); m.szeremeta.23@gmail.com (M.S.); leon.klimont@gmail.com (P.K.); mar.2.ta@wp.pl (M.K.-R.); wojtom67@gmail.com (T.W.); 2 Department of General, Oncological and Vascular Surgery, 5th Military Clinical Hospital in Kraków, 30-705 Krakow, Poland; tomasz.stefura@gmail.com; 3 Department of Medical Education, Faculty of Medicine, Jagiellonian University Medical College Kraków, 30-688 Krakow, Poland; 4 Scientific Editorial Office, Maria Skłodowska-Curie Memorial, National Institute of Oncology, Roentgena 5, 02-781 Warsaw, Poland

**Keywords:** melanoma, sentinel node biopsy, complete lymph node dissection, lymphadenectomy, meta-analysis

## Abstract

The aim of this meta-analysis was to answer the question as to whether performing CLND (complete lymph node dissection) is necessary in every case of the melanoma patient after the positive SNB (sentinel node biopsy). To resolve doubts the authors reanalyzed previous articles and systematized the knowledge about the concerning medical problem. The databases such as PubMed, Scopus and Web of Science were screened to find articles that will be helpful to answer the controversial question if performing lymphadenectomy is crucial. The inclusion criteria consisted of randomized clinical trials, comparison of lymphadenectomy versus observation and positive sentinel node biopsy. After which, seven articles were examined. Authors analyzed parameters such as: recurrence, 3-year survival and 5-year survival. There was no relationship between the performance of CLND and melanoma recurrence (OR 1.04; 95% CI: 0.82–1.31; *p* = 0.75). However, no CLND group had higher 3-year survival (OR 1.22; 95% CI: 1.03–1.44; *p* = 0.02) and 5-year survival (OR 1.30; 95% CI: 1.19–1.85; *p* = 0.008). In conclusion, the observational approach to the melanoma patients with positive sentinel node biopsy is associated with comparable or slightly improved 3- and 5-year survival, then in case of routine lymphadenectomy. Although, in each melanoma patient a decision to perform or withhold lymphadenectomy should always be considered individually. Patients with low perioperative risk could be considered for surgical approach. The study was registered in PROSPERO and was assigned with the unique identifying number “CRD42021241272”.

## 1. Introduction

According to the definition, melanoma is a malignant neoplasm originating from melanocytes dispersed in the skin, mucosa and other organs. Recently there has been a rapid increase in worldwide incidence of new melanoma cases reaching 132,000 globally each year [1].

Early melanoma management for over last two decades included wide surgical excision with clear histological margins followed by examination of the sentinel node (the first draining lymph node to be affected by metastatic disease) to detect clinically occult disease. In sentinel lymph node positive cases, completion lymph node dissection (CLND) was performed as a standard of care. Nevertheless, two major studies published recently, which led to the update of the current guidelines, showed a lack of up-front CLND efficacy as compared to ultrasonographic observation of axilla [2,3,4,5].

The current management of melanoma is based on the patient’s qualification for a sentinel node biopsy (SNB). Patients qualified for SNB are patients who: have excision biopsy and have histopathologic confirmation of melanoma, do not show clinical signs of both distant and regional metastases, with Breslow ≥ 0.8 mm or with ulceration on the lesion surface, regardless of the Breslow parameter. In some cases of pT1b melanomas with a thickness of 0.8–1.0 mm, when unfavorable prognostic factors coexist, SNB can be performed without relying on previous criteria [6]. According to NICE 2022 and ESMO 2020 guidelines SNB should be considered in staging of >1.0 mm Breslow thickness in melanoma of AJCC8 stage pT2a, IB, IIC or higher. Melanomas of AJCC8 stage pT1b, with any of the risk factors such as ulceration or high mitotic rate, are questionable—SNB should be taking under advisement [7,8].

The purpose of this meta-analysis was to provide a high-quality and most up-to-date comparison of lymphadenectomy and observational approach in melanoma. In addition to the previous meta-analyses, we designed our search strategy wider, to include all possibly relevant studies published so far and to systematize available knowledge.

## 2. Materials and Methods

The study was designed to compare two therapeutic options in melanoma patients with positive sentinel lymph node biopsy (SLNB): CLND vs. observation. This systematic review and meta-analysis has been registered in PROSPERO, and was assigned with the unique identifying number “CRD42021241272” [9]. Ethical approval and the informed consent of the participants are not required in systematic reviews with meta-analysis.

### 2.1. Search Terms

The authors followed the PRISMA guidelines to obtain the highest quality of the work (Supplementary Materials) [10]. The search was conducted using PubMed, Scopus and Web of Science databases and it was limited to studies published between 1995 and 2021. The search strategy was based on following terms: “melanoma”, “malignant melanoma”, “lymphadenectomy”, “complete lymph node dissection”, “sentinel node biopsy”, “positive sentinel node biopsy”, “lymphatic metastasis”, “randomized controlled trial”, “humans” and “compared with”. All above mentioned terms were combined using operators such as “AND” and “OR”. All found abstracts were screened for inclusion by members of the review team (KR, KM, JT, JK, MS and PK), supervised by senior authors (TS, MKR, TW and WMW). Selected abstracts were assessed profoundly and included in meta-analysis correspondingly to the inclusion criteria.

### 2.2. Eligibility Assessment

The authors conducted an eligibility assessment for all the full-text articles that were filtered during the abstract screening process. We have included randomized clinical trials that reported the treatment of melanoma patients with positive sentinel node biopsy and compared the complete lymph node dissection versus observational approach (i.e., surgery vs. no surgery). Furthermore, the articles had to be written in English. The authors excluded all meta-analyses, systematic reviews and otherwise irrelevant studies.

### 2.3. Data Extraction

Six reviewers recorded relevant data from included manuscripts. At least two authors extracted data from each article. If any conflicting data occurred, the study was subjected to further discussion with one of senior authors.

### 2.4. Outcomes of Interest

Data were isolated from included studies: first author, publication year, title, type of the study, country, follow up in months, sex, mean or median age and sample; sample divided into groups: CLND and no CLND; tumor location: head, trunk, extremities, ulceration, Breslow depth, deaths and recurrence; HR: 3-year survival, disease free survival, 5-year survival, melanoma specific survival, overall survival and recurrence free survival.

### 2.5. Quality Assessment

Quality of the included articles was assessed using the Cochrane risk-of-bias tool for randomized trials (RoB 2). There were several parameters that were measured, such as: “Randomization process”, “Deviations from the intended interventions”, “Missing outcome data”, “Measurement of the outcome” and “Selection of the reported result”. Each article was assessed as “high risk”, “low risk” and “some concerns”.

### 2.6. Statistical Analysis

Statistical analysis was performed using Review Manager 5.4 (The Cochrane Collaboration, 2020, London, UK). The authors analyzed three parameters: recurrence, 3-year survival and 5-year survival, next they divided articles into 3 subgroups. Statistical parameters such as odds ratios (OR) were generated with 95% confidence intervals (CI) and statistical significance reached to 0.05. Heterogeneity of studies was evaluated using I2 test and value above 70 constituted considerable heterogeneity. The quality of analyzed works was checked using Cochrane risk-of-bias tool for randomized trials.

## 3. Results

### 3.1. Articles Selection

**Figure 1:**
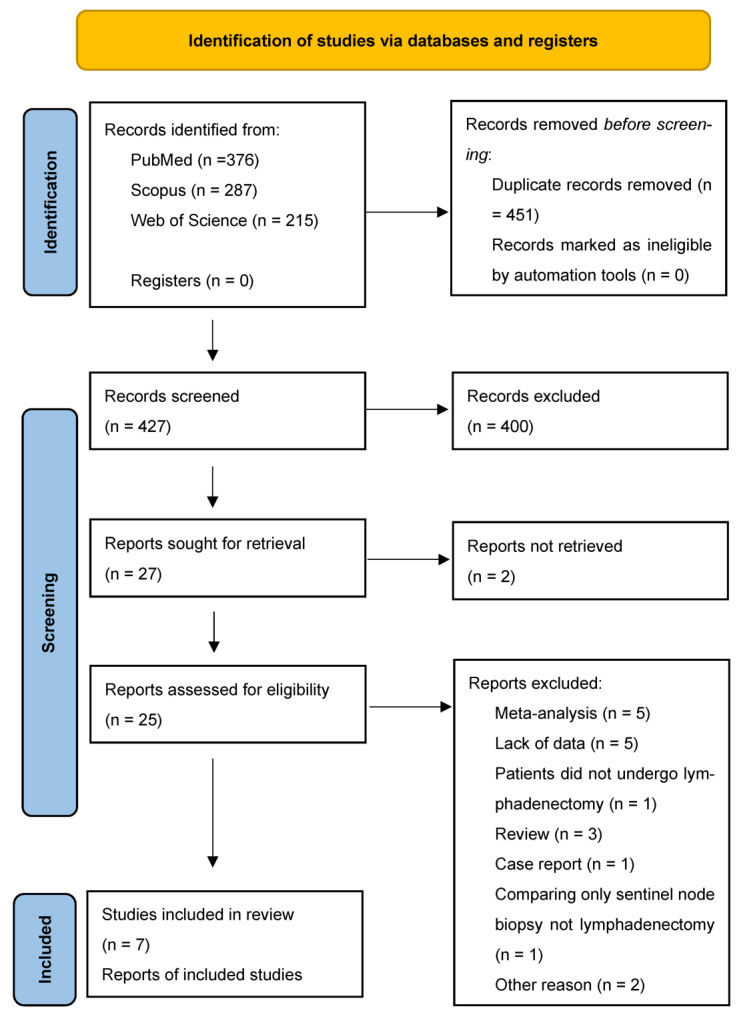
PRISMA flow-diagram of the study inclusion process.

Primary search strategy produced 744 records, after which qualification process followed, as well as elimination of duplicates, resulting in 425 articles, of which 22 datasets met the criteria for full-text review [2,3,11,12,13,14,15,16,17,18,19,20,21,22,23,24,25,26,27]. Finally, seven papers were included in the meta-analysis (Table 1). The details of the process have been stated in the PRISMA flow-diagram (Figure 1).

### 3.2. Articles Characteristic

Seven articles were included into the statistical analysis [3,4,28,29,30,31,32]. Chosen articles contained patients treated in Europe and USA between 1995 and 2016. We stratified data into three subgroups to assess the following outcomes measures: (1) “Melanoma Recurrence”, (2) “3-year survival” and (3) “5-year survival”.

### 3.3. Patients Characteristics

The authors included seven articles and a total group of 5321 patients were analyzed. There were 3711 patients who underwent CLND and 1610 patients in whom further surgery was omitted. Complete datasets for given outcomes measures included in total: 1922 patients for melanoma recurrence, 3403 patients for 3-year survival and 3093 patients for 5-year survival.

### 3.4. Melanoma Recurrence

**Figure 2:**
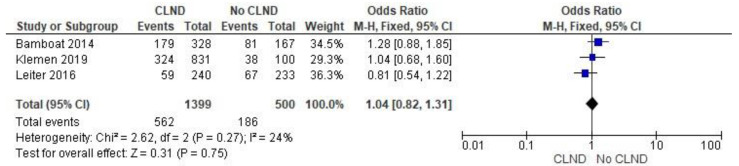
Melanoma recurrence [28,29,31].

Meta-analysis of outcomes presented by Bamboat et al., Klemen et al., and Leiter et al. did not prove a statistically significant relationship between the performance of CLND and melanoma recurrence (OR 1.04; 95% CI: 0.82–1.31; *p* = 0.75). The results are presented in Figure 2.

### 3.5. Three-Years Melanoma Survival

**Figure 3:**
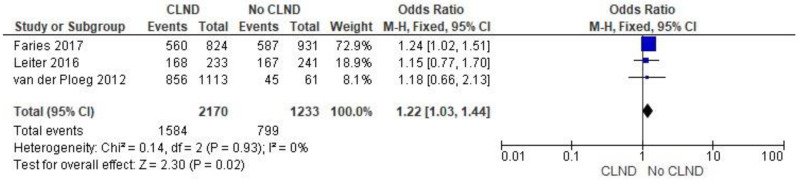
Three-years melanoma survival [4,31,32].

The analysis of 3-year overall survival included the three studies (Faries et al., Leiter et al., Van der Ploeg et al.). It revealed a statistically significant difference favoring no CLND approach (OR 1.22; 95% CI: 1.03–1.44; *p* = 0.02). The results are presented in Figure 3.

### 3.6. Five-Years Melanoma Survival

**Figure 4:**
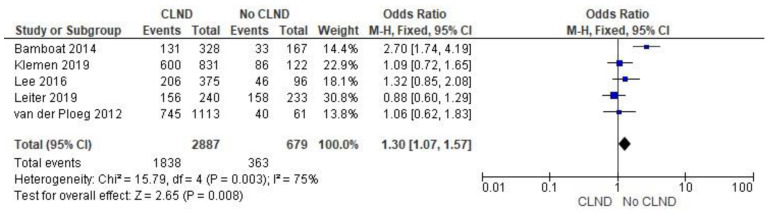
Five-years melanoma survival [3,28,29,30,32].

Five-years melanoma survival analysis included five articles (Bamboat et al., Klemen et al., Lee et al., van der Ploeg et al. 2012, Leiter et al.). Patients undergoing a no CLND approach had higher odds of overall 5-year survival (OR 1.30; 95% CI: 1.19–1.85; *p* = 0.008). The results are presented in Figure 4.

### 3.7. Quality Assessment

**Figure 5:**
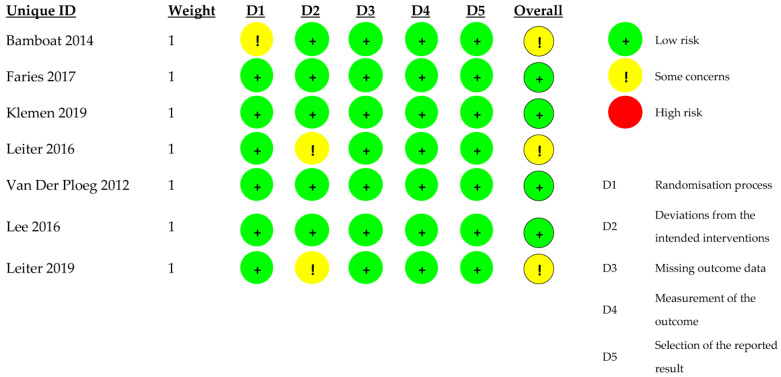
Quality assessment—Cochrane risk-of-bias tool for randomized trials (RoB 2) [3,4,28,29,30,31,32].

There was a low risk for bias in the “Missing outcome data”, “Measurement of the outcome” and “Selection of the reported result” domains. There were some concerns involving the risk of bias in domains “Randomization process” and “Deviations from the intended interventions”. The quality of the included articles is presented in Figure 5.

## 4. Discussion

In the past, melanoma patients have routinely undergone elective dissection of the lymph nodes, which was associated with all variety of well-known potential complications [33]. In case of performing CLND we can expect occurrence of lymphorrhea, sensory disturbances and lymphoedema [34]. Prophylactic lymphadenectomy is not recommended due to lack of the therapeutic benefit. It can be considered in the presence of lymph node metastases to avoid further dissemination of the disease and to achieve locoregional control. Patients with a tumor diameter <0.1 mm should not undergo routine lymphadenectomy, whereas tumor-diameter of 0.1 mm and 1 mm and above, a total lymphadenectomy may be offered. [35]. Nowadays according to NICE 2022 guidelines therapeutic CLND should be performed in melanoma patients with clinical stage IIIB-IIIC or in any patient that has nodal changes detected in imaging tests [7]. The ESMO 2020 guidelines also support these recommendations [8]. According to German guidelines, the risk of a positive sentinel node correlates with the presence of ulcers, tumor size and a high rate of mitosis. [35]

One of the most important concepts that changed the therapeutic approach of patients with melanoma was the introduction of sentinel node biopsy prior to lymphadenectomy, which nowadays is the standard of care [14,36,37]. The SNB is a key element in planning the therapeutic procedure—it determines the need for lymphadenectomy, qualification for adjuvant treatment after the procedure and allows for the control of disease advancement in regional lymph nodes [6,36,37,38,39].

The aim of our metanalysis was to assess, whether lymphadenectomy should be performed in every case of melanoma patient with positive sentinel node biopsy. After many years, without an unequivocal answer, studies were designed to reanalyze this issue. The value of our pooled study consists in scrupulous analysis of a large group of relevant studies and integrating them in such a way as to draw a unified conclusion. Furthermore, search strategy was wider as compared to previously attempted pooling of data [14,15,17,18,23,40].

According to our results there was no statistically significant difference between CLND and noCLND groups in terms of melanoma recurrence. Nevertheless, when analyzing the 3-year and 5-year survival outcomes favored the group in which the lymphadenectomy was not performed. Additionally, Breit et al., after analyzing works of other authors, suggests that performing a lymphadenectomy does not increase melanoma specific survival compared to patients in whom lymphadenectomy was abandoned [4,12,31,33,40,41]. The study of Standage (2019) showed that observational approach is the most efficient from among therapeutic options in melanoma patients with positive SNB. The advantage of the observational approach over lymphadenectomy results not only from the greater benefits for the patient’s quality of life, higher 3- and 5-year survival, but also from the lower costs [42].

There are several potential sources of heterogeneity in this study. Although the study design and data collection was conducted correctly there is a risk of high heterogeneity of the study group which can form a problem when comparing parameters. The observed variability is due to differences at the clinical level of melanoma—in included studies there was no division into types of recurrence (locoregional or distal).

When making decisions concerning CLND, all circumstances should be considered, especially the potential benefits for the patients and potential risks associated with surgery [14]. However, if a lymphadenectomy is not performed, the patient should be monitored using ultrasonography of regional lymph nodes every 3–4 months, which requires high level of compliance [35,43,44].

## 5. Conclusions

Therefore, in each case the decision to perform or withhold lymphadenectomy in patients with melanoma and a positive sentinel node, should be considered individually and taken after profound discussion with the patient. The observational approach to the melanoma patients with positive sentinel node biopsy is associated with comparable or even slightly improved survival. Patients with low perioperative risk could be still considered for surgical approach, particularly if no strict clinical follow-up is available to them.

## Figures and Tables

**Table 1:** Articles included in the meta-analysis.

First Author, References	Publication Year	Title	Study Design	Country	Follow-Up in Months	Sex (% of Women)	Mean or Median Age	Sample (*n*)	Sample—Clnd	Sample—no CLND	Tumor Location Head (*n*)	Tumor Location Head (%)	Tumor Location Trunk (*n*)	Tumor Location Trunk (%)	Tumor Location Extremities (*n*)	Tumor Location Extremities (%)	Ulceration (*n*)	Ulceration (%)	Breslow [mm]	Deaths (*n*)	Deaths (%)	Recurrence (*n*)	Recurrence (%)
Klemen, N.D. [28]	2019	Completion lymphadenectomy for a positive sentinel node biopsy in melanoma patients is not associated with a survival benefit	retrospective	International	28.9	39	54.5	953	831	122	126	13	397	42	414	43	120	13	2.5	227	28	324	39
Bamboat, Z.M. [29]	2014	Observation after a Positive Sentinel Lymph Node Biopsy in Patients with Melanoma	retrospective	International	66	37.7	66	495	167	328	x	x	x	x	x	x	x	x	x	x	x	260	52.5
Lee, D.Y. [30]	2016	Impact of completion lymph node dissection on patients with postive sentinel lymph node biopsy in Melanoma	retrospective	USA	83.1	33.3	57	471	375	96	24	22.2	x	x	49	45.4	x	x	x	x	x	x	x
Leiter, U. [31]	2016	Complete lymph node dissection versus no dissection in patients with sentinel lymph node biopsy positive melanoma (DeCOG-SLT): a multicentre, randomised, phase 3 trial	rct	Germany	35	38.5	54	473	240	233	x	x	247	52	31	13	95	41	x	44	14	67	29
Faries, M.B. [4]	2017	Completion Dissection or Observation for Sentinel-NodeMetastasis in Melanoma	rct	International	43	41.5	53	1755	824	931	241	13.73	805	45.7	709	40.4	894	50.9	x	x	x	x	x
van der Ploeg, A.P.T. [32]	2012	Prognosis in patients with sentinel node-positive melanoma without immediate completion lymph node dissection	retrospective	International	34	47.5	55	1174	1113	61	154	13.1	481	41	526	44.8	529	45.1	2.5	x	x	x	x
Leiter, U. [3]	2019	Final Analysis of DeCOG-SLT Trial: No Survival Benefit for Complete Lymph Node Dissection in Patients with Melanoma with Positive Sentinel Node	rct	Germany	72	x	54	483	242	241	x	x	x	x	x	x	185	38.3	2.4	133	27.5	166	34.4

x—Lack of data in the article.

## Data Availability

Not applicable.

## Supplementary Materials

The following supporting information can be downloaded at: https://www.mdpi.com/article/10.3390/jcm11133880/s1.
